# Relative Prognostic Value of Human Epidermal Growth Factor Receptor 2 (HER2) Expression in Operable Oesophagogastric Cancer

**DOI:** 10.5402/2012/804891

**Published:** 2012-07-26

**Authors:** David S. Y. Chan, Fiona Campbell, Paul Edwards, Bharat Jasani, Geraint T. Williams, Wyn G. Lewis

**Affiliations:** ^1^Department of Surgery, University Hospital of Wales, Heath Park, Cardiff, CF14 4XN, UK; ^2^Department of Cellular Pathology, University Hospital of Wales, Heath Park, Cardiff CF14 4XN, UK; ^3^Department of Surgery, Nevill Hall Hospital, Abergavenny NP7 7EG, UK; ^4^Institute of Cancer and Genetics, Cardiff University School of Medicine, Cardiff CF14 4XW, UK

## Abstract

*Aims*. The aim of this study was to determine the prognostic significance of HER2 receptor expression in operable oesophagogastric adenocarcinoma. *Methods*. Eighty-five consecutive patients diagnosed with oesophagogastric adenocarcinoma [18 oesophageal (OC), 32 junctional (JC) and 35 gastric (GC)] undergoing potentially curative resection were studied retrospectively. Immunohistochemistry was used to determine HER2 status at endoscopic biopsy and resection specimen. The primary outcome measure was survival. *Results*. Twenty (24%) patients had HER2 positive tumours which was commoner in JC (14/32, 44% versus 2/18, 11% in OC and 4/35, 11% in GC, *P* = 0.003). The sensitivity, specificity, positive and negative predictive values of HER2 status at endoscopic biopsy were 56%, 93%, 63%, 91% respectively (weighted Kappa = 0.504, *P* < 0.0001). Five-year survival in OC HER2 positive negative was 100% and 36% (*P* = 0.167) compared with 14% and 44% (*P* = 0.0726) in JC and 50% and 46% (*P* = 0.942) in GC respectively. *Conclusions*. Endoscopic biopsy had a high specificity and negative predictive value in determining HER2 status. Patients with JC had a significantly higher rate of HER2 overexpression and this was associated with a nonsignificant poorer survival trend. A larger study is needed to confirm these findings because of the implications for neoadjuvant and adjuvant chemotherapy regimens.

## 1. Introduction

The worldwide burden of oesophagogastric cancer is growing. Each year 482,300 and 989,600 people are diagnosed with oesophageal and gastric cancer resulting in 406,000 and 738,00 deaths, respectively [1]. The optimal contemporary treatment is controversial and opinion divided. In oesophageal cancer, following the publication of the MRC OEO2 trial, neoadjuvant chemotherapy followed by surgery is the standard of care for patients with operable oesophageal cancer [2] in the United Kingdom, whereas neoadjuvant chemoradiotherapy followed by surgery is the preferred modality in Europe and the United States [3]. Moreover, the optimum treatment for patients diagnosed with gastric cancer in the UK remains controversial, both in terms of neoadjuvant chemotherapy [4] and the extent of the lymphadenectomy. However, overall survival reports remain poor and no established global standard for treatment exists. New therapies which target specific genetic alterations arguably offer the best chance for improving patient survival.

The human epidermal growth factor receptor 2 (HER2) gene is a protooncogene which is located on chromosome 17q11.2–12 and encodes a transmembrane tyrosine kinase receptor which is responsible for cell growth, differentiation, migration, and apoptosis [5]. HER2 is involved in the development of numerous types of cancer and is overexpressed in up to 25% of breast cancer patients, conferring a poor prognosis [6]. In oesophagogastric cancer, HER2 overexpression has been reported at frequencies similar to those observed in breast cancer, ranging from 16% to 27% [7–10].

A combination of the monoclonal antibody against HER2 (trastuzumab) with standard chemotherapy improved survival significantly in patients with HER2 positive advanced gastric cancer in the Trastuzumab for Gastric Cancer (ToGA) trial [11]. All patients in this trial had inoperable junctional or gastric adenocarcinoma and there is currently no evidence for the use of trastuzumab in operable HER2 positive oesophagogastric cancer in the neoadjuvant setting prior to surgery.

The relationship between HER2 overexpression and prognosis in operable oesophgogastric cancer is controversial [12, 13]. Some studies have suggested that HER2 overexpression is associated with poor survival in oesophageal [14, 15] and gastric cancer [7, 10], whereas others have shown no association with prognosis [8, 16–19].

The primary aim of this study was therefore to determine the prognostic significance of HER2 overexpression in patients with operable oesophagogastric adenocarcinoma. The secondary aim was to determine the accuracy of the endoscopic index biopsy in assessing HER2 overexpression when compared with the final operative resection specimen.

## 2. Methods

Eighty-five consecutive patients diagnosed with oesophagogastric adenocarcinoma (18 oesophageal (OC), 32 junctional (JC), and 35 gastric (GC)) undergoing R0 resection between 1 February 2001 and 30 June 2006 were studied retrospectively. All tumours were staged in accordance with the International Union against Cancer tumour node metastasis (TNM) classification of malignant tumours TNM6 [20]. The primary outcome measure was survival from diagnosis. Ethical approval was obtained from the local ethics committee.

### 2.1. Neoadjuvant Chemotherapy

The selective use of neoadjuvant chemotherapy was adopted in the latter part of the study period and was given to 25 patients with minimal comorbidities who were deemed to have relatively advanced disease and would benefit from downstaging of the tumour prior to surgery. Chemotherapy was administered for three or four cycles preoperatively and postoperatively. Each cycle consisted of epirubicin (50 mg/m^2^) by intravenous bolus, cisplatin (60 mg/m^2^) as a four-hour infusion on day 1 and 5-fluorouracil (200 mg/m^2^/day) daily by a continuous intravenous infusion.

### 2.2. Surgical Treatment

Patients with oesophageal cancer were selected for radical treatment based on perceived radiologic stage, comorbidity, and patient choice according to algorithms described previously [21–23]. The type of surgery for gastric cancer was determined by the anatomical location of the tumour; subtotal gastrectomy was performed in patients with antral tumours and total gastrectomy was performed in tumours of the cardia (Siewert type III), body, and linitis plastica. A modified D2 lymphadenectomy preserving the spleen and pancreas was performed [24].

### 2.3. Immunohistochemistry

Immunohistochemistry (IHC) was used to determine patients' HER2 status at the endoscopic index biopsy and the final operative resection specimen. Sections (4 *μ*m) of tissue were cut, mounted on coated slides, labeled, and then placed on the Ventana Benchmark XT (Roche Diagnostics) for detection of the HER2 oncoprotein. The sections were dewaxed then subjected to pretreatment with CC1 for 30 minutes. Sections were then washed with reaction buffer followed by incubation with the rabbit monoclonal primary antibody HER2/neu (clone 4B5, Pathway) for 16 minutes. On board detection using ultraView Universal DAB kit (Roche Diagnostics), used in accordance with the manufacturer's recommendations, was used to detect the location of the primary antibody HER2 followed by counterstain with haematoxylin II for 4 minutes (Roche Diagnostics). 

All sections were reviewed independently by two consultant histopathologists (Jassani and Williams) who were blinded to all clinical and pathological information. Discordant cases were reviewed together and a final consensus was reached. Evaluation and scoring of HER2 protein overexpression was performed according to the Dako HercepTest scoring system for breast cancer. Only membranous staining was considered. This scoring system has been validated for use in gastric cancer with minimal modifications: 0/negative = staining or membranous reactivity in <10% of cells, 1+/negative = faint membranous reactivity in >10% of cells or cells with reactivity only in part of their membrane, 2+/equivocal = weak/moderate complete or basolateral membranous staining in >10% of tumour cells; and 3+/positive = strong complete or basolateral membranous staining in >10% of tumour cells [25, 26].

### 2.4. Follow-Up Evaluation

Patients were reviewed every 3 months for the first year, then every 6 months thereafter. The median follow-up period was 71 months. A total of 79 patients (93%) were followed up for at least 5 years or until death. Death certification was obtained from the Office for National Statistics.

### 2.5. Statistical Analysis

Statistical analysis appropriate for nonparametric data was used. Grouped data were expressed as median (range). Groups were compared with the Mann-Whitney *U* test for unpaired data. The agreement between HER2 status at index biopsy and the postoperative histopathological resection specimen was determined using the weighted Kappa statistic (Kw) [27]. The value of Kappa has a maximum of 1.00 when agreement is perfect, a value of zero indicates no agreement better than chance and negative values show worse than chance agreement. The strength of agreement was assessed according to the guidelines of Landis and Koch [27]. The sensitivity and specificity, positive predictive value and negative predictive value were also estimated. Cumulative survival was calculated by the life table method of Kaplan and Meier [28]. Differences in survival times between groups of patients were analysed by the log rank method [29]. Multivariate Cox regression was used to assess the prognostic value of individual variables. Data analysis was carried out with the Statistical Package for Social Sciences (SPSS) version 18 (SPSS, Chicago, Illinois, USA).

## 3. Results

Twenty (24%) patients had HER2 positive tumours, and positive HER2 status was commoner in JC (14/32, 44% versus 2/18, 11% in OC and 4/35, 11% in GC; *χ* ^2^ = 11.66, *P* = 0.003), Table 1.

### 3.1. Accuracy of Biopsy in Determining HER2 Status

Comparison of HER2 expression status between the index biopsy and final operative resection specimen revealed sensitivity, specificity, and positive and negative predictive values were 56%, 93%, 63%, 91%, respectively. There was strong agreement between the index biopsy and final operative resection specimen (weighted Kappa statistic was 0.504; 95% CI 0.128–0.856; *P* < 0.0001). Only 3 patients had a false positive biopsy result, all of whom had undergone gastrectomy without neoadjuvant chemotherapy.

### 3.2. Outcomes Related to HER2 Expression

**Figure 1:**
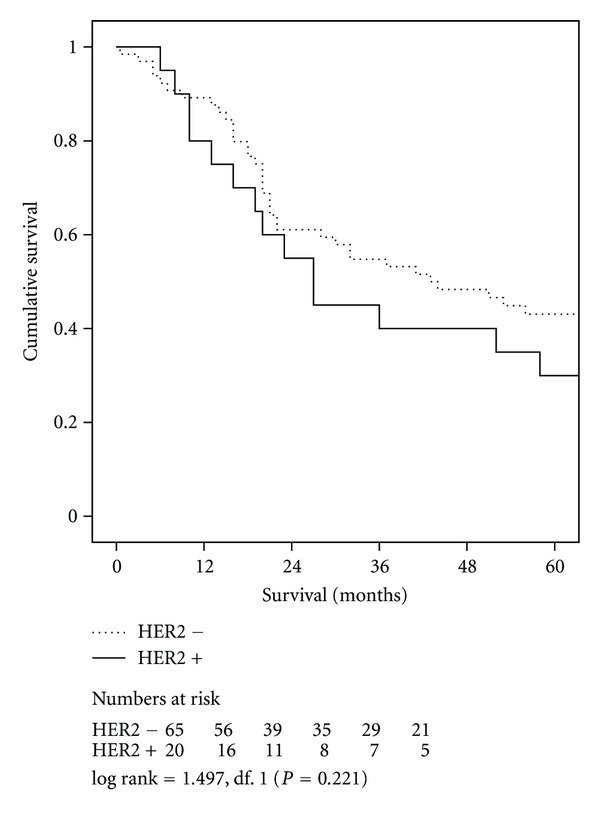
Survival related to HER2 overexpression in *all patients. *

**Figure 2:**
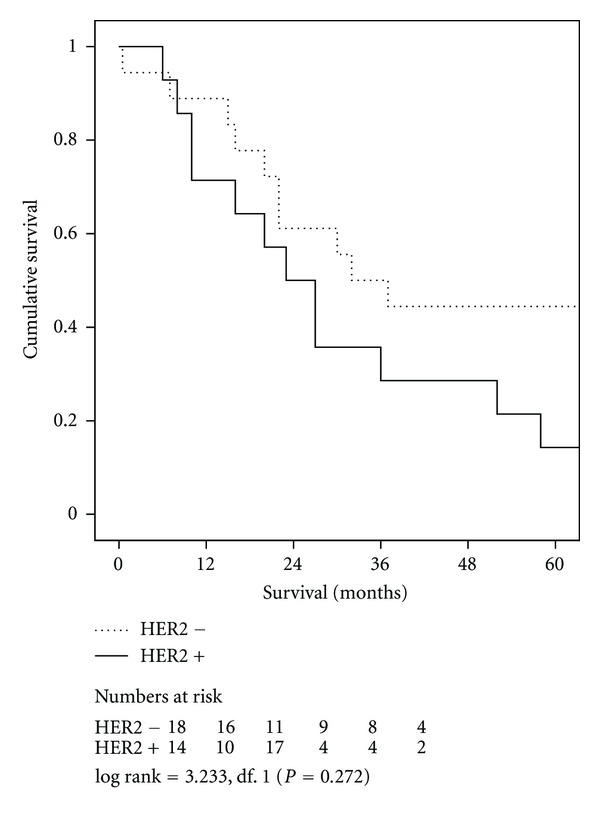
Survival related to HER2 overexpression in patients with *junctional adenocarcinoma. *

Short-term outcomes were similar in patients with HER2 positive and negative tumours (Table 2). Cumulative five-year survival related to HER2 status was 30% for the HER2 positive cohort compared with 43% for the HER2 negative cohort (*P* = 0.221), (Figure 1). With regard to tumour site, 5-year survival in OC HER2 positive versus negative cohorts was 100% and 36% (*P* = 0.167) compared with 14% and 44% (*P* = 0.0726) in JC (Figure 2) and 50% and 46% (*P* = 0.942) in GC, respectively. Univariate analysis of factors associated with duration of survival is shown in Table 3.

## 4. Discussion

HER2 overexpression was found in 24% of patients in this cohort, the majority of whom had tumours situated around the oesophagogastric junction. This is in keeping with recent studies reporting a prevalence rate of 15–30% [8, 11, 14]. The previously quoted range of 11–73% largely originates from studies conducted in the 1990s which adopted various cutoffs for the classification of HER2 status preventing valid comparisons to be made [15, 30–32]. There was a trend towards poorer long-term survival in patients diagnosed with HER2 positive operable oesophagogastric cancer compared to patients with HER2 negative tumours. However, this difference was not statistically significant and this finding echoes more recent reports which have also shown that HER2 overexpression was not associated with duration of survival [8, 12, 17, 33].

The accuracy of endoscopic biopsy in determining HER2 status in oesophagogastric cancer has not been documented previously. We report a high specificity and negative predictive value of endoscopic biopsy in determining HER2 status in oesophagogastric cancer. However, the sensitivity and positive predictive value was lower when compared with breast core-needle biopsies [34]. The pattern of HER2 staining in breast cancer tends to be homogenous, whereas HER2 staining in gastric cancer is heterogeneous [26]. A modified scoring system which only takes into account the pattern of reactivity irrespective of the number of reactive cells in biopsy specimens has therefore been introduced [26].

This study has several potential limitations. This was a retrospective observational study and is therefore open to selection bias. The relatively small sample size could have resulted in a type II error. HER2 status was determined using IHC on tissue samples which have been stored for a few years prior to analysis. Deterioration in antigenicity can occur once sections from paraffin blocks have been put onto slides [35] and could arguably underestimate the prevalence of HER2 overexpression in our cohort. However, this appears to be a limitation of most studies published on this subject [8, 16, 36]. Although the assessment of HER2 gene amplification with in situ hybridisation (ISH) techniques has been recommended to determine the final HER2 status in equivocal IHC 2+ [26], we did not perform ISH on the 5 patients in our cohort who had IHC 2+ as the concordance between IHC and ISH have been shown to be high [26, 37].

Conversely, the strengths of the study are that the demographic data and outcomes were collected prospectively, from a well-defined geographical area served by an established regional upper GI network. The study's survival and prognostic data are especially robust because no patients were lost to followup, and causes and exact dates of death were obtained from death certificates provided by the Office for National Statistics. HER2 status was determined by two specialist consultant histopathologists, one of whom (B.J) was part of the steering group recommending guidelines for HER2 testing in the UK [25, 38, 39].

A larger prospective study using validated and reproducible methods in IHC and ISH is needed to clarify the prognostic role of HER2 in patients with operable oesophagogastric cancer. The accuracy of the index biopsy at determining HER2 status is important as the addition of anti-HER2 therapy to the standard neoadjuvant chemotherapy regime may be beneficial in HER2 positive patients. Future studies into targeted molecular therapies should also take into account characteristics of both the primary tumour and disseminated tumour cells [8].

## 5. Conclusions

Endoscopic biopsy had a high specificity and negative predictive value in determining HER2 status. Patients with JC had a significantly higher rate of HER2 overexpression and this was associated with a nonsignificant poorer survival trend. A larger study is needed to confirm these findings because of the implications for neoadjuvant and adjuvant chemotherapy regimens.

## Figures and Tables

**Table 1:** Details of patients.

	OC	JC	GC	*P* value
Number	18	32	35	
Median age (years)	60	66	72	0.012
Gender M : F (%)	18 : 0 (100 : 0)	27 : 5 (84 : 16)	19 : 16 (54 : 46)	<0.0001
Surgery (%)				
TTO	7 (39)	9 (28)	—	
THO	11 (61)	13 (41)	—	
TG	—	10 (31)	9 (26)	
STG	—	—	26 (74)	
HER2+ (%)	2 (11)	14 (44)	4 (11)	0.003
pTNM (%)				
I and II	10 (56)	12 (37)	20 (57)	0.062
III and IV	8 (44)	20 (63)	15 (43)	

OC: Oesophageal adenocarcinoma; JC: junctional adenocarcinoma; GC: gastric adenocarcinoma; TTO: transthoracic oesophagectomy; THO: transhiatal oesophagectomy; TG: total gastrectomy; STG: subtotal gastrectomy.

**Table 2:** Outcome related to HER2 overexpression.

	HER2 −	HER2+	*P* value
Number (%)	65 (76)	20 (24)	
Median age (years)	66	69	0.705
Gender M : F (%)	48 : 17 (74 : 26)	16 : 4 (80 : 20)	0.577
pTNM (%)			
I and II	(52)	(40)	0.089
III and IV	(48)	(60)	
Morbidity (%)	23 (35)	7 (35)	0.975
Mortality (%)	2 (3)	1 (5)	0.47
Median survival (months)	43	27	0.221
1 year survival (%)	89	80	
2 year survival (%)	61	55	
5 year survival (%)	43	30	

**Table 3:** Univariate analysis of factors associated with duration of survival.

Factor	*χ* ^2^	df	*P* value
HER2 overexpression	1.497	1	0.221
pT stage	17.346	3	0.001
Age	68.826	4	0.001
pN stage	34.272	3	<0.0001
pTNM stage	30.786	3	<0.0001
Lymph node ratio	183.926	3	<0.0001
